# Telemedicine Use, Comfort, and Perceived Effectiveness in the Spinal Muscular Atrophy Community

**DOI:** 10.1089/tmj.2023.0293

**Published:** 2024-02-07

**Authors:** Ilse S. Peterson, Lisa T. Belter, Mary A. Curry, Jill Jarecki

**Affiliations:** ^1^ Faegre Drinker Biddle and Reath, LLP, Washington, District of Columbia, USA.; ^2^ Milken Institute School of Public Health, The George Washington University, Washington, District of Columbia, USA.; ^3^ Cure SMA, Elk Grove, Illinois, USA.

**Keywords:** spinal muscular atrophy, telemedicine, teleneurology, patient experience

## Abstract

**Background:**

Telemedicine may increase access to clinical care, particularly for mobility-limited communities such as the spinal muscular atrophy (SMA) community. However, much of the information on exposure to and attitudes toward telemedicine in neuromuscular diseases generally and SMA specifically is anecdotal or from focus groups. Gaining greater insight into patient perspectives is important, given telemedicine's potential for expanding access to care and growing use of telemedicine as a result of technology advances and the COVID-19 pandemic.

**Methods:**

Cure SMA collected information on the SMA community's exposure to, comfort with, and perceived effectiveness of telemedicine through its 2021 Community Update Survey. The final analytic sample represented 463 SMA-affected individuals, resident in the United States. Descriptive analyses, correlations, and ordered logit regression models were used to characterize the sample and identify predictors of exposure, comfort, and perceived effectiveness. Data were analyzed on weighted and unweighted bases to account for differences between the survey sample and the SMA community. Stratified analyses were used to compare self-completed surveys with caregiver-completed surveys.

**Results:**

463 individuals answered questions about telemedicine. Approximately four-fifths of these respondents had used telemedicine previously. Factors predicting greater likelihood of prior telemedicine use included male gender, increasing income, having received drug treatment for SMA, history of mental illness, and having non-neutral views regarding comfort and perceived effectiveness of telemedicine. Several factors were also significant predictors of comfort with and perceived effectiveness of telemedicine. Stratified analyses indicated differences between self-completed and caregiver-completed surveys.

**Conclusion:**

These results can provide insight into patient experiences with telemedicine and can inform approaches to its use by health care professionals and clinical trial sponsors.

## Introduction

Interest in telemedicine has grown substantially in recent years, with uptake accelerated by the COVID-19 pandemic and changes to reimbursement policies.^1–5^ Telemedicine holds special interest for neuromuscular disease communities because of its potential to mitigate mobility-associated challenges to health care access, and has been shown in a variety of disease settings to be an effective platform for delivering care and monitoring patients.^5–11^ It also holds promise for improving access to care for individuals who are geographically distant from specialists, from historically underserved communities, or otherwise unable to travel.^5,12^ Finally, telemedicine may provide a cost-effective means of improving care continuity and—in research settings—facilitate patient recruitment and retention, while reducing direct and indirect costs related to patient transfers and travel.^9,12–14^

While telemedicine holds considerable potential, its use in neuromuscular settings poses unique challenges. Management of neuromuscular diseases—including spinal muscular atrophy (SMA), which is the focus of this research—often requires comprehensive examinations and care from multidisciplinary teams.^7,15^ Furthermore, many motor function outcome measures that assess muscle, strength, and reflexes are designed for in-person settings.^6,8,16^ In addition to being important for assessing health status, these measures may be required for coverage of health care services and drug treatments. For these reasons, telemedicine is expected to play a growing role in neuromuscular disease care, but is unlikely to fully supplant in-person care.

SMA is an area in which there is particular interest in telemedicine. SMA is a progressive neuromuscular disorder with an estimated live birth incidence of 1: 10,000, and one of the most common genetic causes of death in infants.^15,17–20^ SMA has been historically classified into types based on age at symptom onset and maximum motor function achieved.

According to historical classifications, individuals with Type 1 typically experience symptom onset before 6 months of age and never gain the ability to sit unsupported. Those with SMA Type 2 have symptom onset between 7 and 18 months and may sit, but will never stand. SMA Type 3 presents after 18 months of age and while individuals can stand and walk independently, they lose these abilities with time. SMA Type 4 is the rarest and least severe, presenting with symptom onset of muscle weakness and motor impairment in the second or third decade of life. In recent years, disease-modifying drugs and newborn screening have dramatically changed the outlook for individuals with SMA, changing disease progression, causing new phenotypes to emerge, and creating the need to rethink the historical approach to disease classification.^15,21^

The broad adoption of newborn screening and availability of disease-modifying therapies for SMA create strong clinical and economic incentives to pursue early intervention and ensure continuity of care, which may be enabled by telemedicine. With 48 states and the District of Columbia conducting newborn screening for SMA as of early 2023, the population of individuals who have been diagnosed with SMA and need access to specialty care is larger and more geographically dispersed than ever.^22^ The availability of effective treatments for SMA heightens the importance of access to care. While the disease burden and economic costs of untreated SMA are substantial, early intervention with approved therapies can significantly improve patient outcomes.^23,24^ Given that the majority of specialists with expertise in SMA are located in major urban centers, telemedicine can play a critical role in connecting patients with care and research opportunities.

Prior research in other disease settings has shown that patients accept telemedicine and report positive experiences with it, but that acceptability and use vary based on factors such as gender, age, and socioeconomic status.^9,25^ Recognizing the potential that telemedicine holds for the SMA community, Cure SMA—the leading United States (U.S.)-based patient advocacy organization for SMA—decided to seek insight into patient perspectives on telemedicine through its 2021 Community Update Survey (CUS). This study utilizes Cure SMA's 2021 CUS data to systematically explore factors that affect the likelihood of telemedicine use, comfort with telemedicine, and perceived effectiveness for use in SMA. Subsequently, this article reflects on implications for telemedicine use.

## Methods

### Dataset

Data on the SMA community's exposure to, comfort with, and perceived effectiveness of telemedicine were derived from the 2021 CUS. This survey collects information on the SMA community's experiences and daily challenges. It is sent annually to the organization's membership database, which is the most comprehensive registry specifically focused on SMA-affected individuals with more than 9,000 affected individuals. The survey is administered through the Web-based platform Alchemer. Eligibility is limited to SMA-affected individuals who have reached the age of majority and caregivers of individuals with SMA who complete the survey on the affected individual's behalf. Caregivers of an individual who has passed away are also eligible.

Before dissemination, the 2021 survey was reviewed and approved by WCG IRB. On April 19, 2021, the link to the online survey was emailed to 6,049 members of the Cure SMA membership database, which matched the eligibility criteria and were opted in to receive the survey. In addition to the email invitations, the survey was posted on the Cure SMA website and social media pages to elicit responses from nonmembers. Survey participants were entered to win one of several incentives for completing a survey. Incentives included registration and hotel packages to the Cure SMA Annual Family Conference, e-gift cards, and handheld game consoles. The survey closed on June 18, 2021, with 929 responses. After incomplete and duplicate surveys (*n* = 268 and *n* = 28, respectively) were removed, the dataset included 633 surveys representing 619 unique individuals.

Before being provided for use in this research, the CUS dataset was cleaned and de-identified by Cure SMA. In instances where affected individuals had more than one survey completed on their behalf (e.g., two parents completed surveys), both surveys were retained in the dataset; however, Cure SMA included a “keep survey” variable to indicate which surveys they deemed more complete and recommended using for analysis. Before analysis, surveys for which the respondent type (affected individual or caregiver) was unknown were dropped, as were surveys for individuals with non-5q types of SMA, for which SMA type was missing, from individuals outside of the United States, and which did not include answers to questions about telemedicine. The final dataset included 463 surveys.

### Analysis

The outcomes of interest for this research included frequency of prior telemedicine use, comfort with telemedicine, and perceived effectiveness of telemedicine. Use was assessed on a 3-point scale and comfort and perceived effectiveness were assessed on 5-point scales. Descriptive analyses and correlations were used to characterize the sample and of its representativeness of the U.S. SMA community. This was done by comparing the sample characteristics with internal Cure SMA community estimates developed using carrier analysis data from Sugarman et al., U.S. census data, and earning reports from SMA drug manufacturers used to estimate the total number of treated individuals nationally.^26^

Chi square tests were used to explore correlations between the outcomes of exposure, comfort, and perceived effectiveness and other factors, including respondent identity; demographic variables; SMA-related factors such as SMA type, mobility, and prior drug treatment; and other variables related to health and health care utilization, including overall health ratings, history of mental illness, insurance coverage, and recent doctor visits.

Ordered logit regression models were used to identify predictors of exposure, comfort, and perceived effectiveness. Unweighted and weighted regression models were run for the full analytic sample as well as stratified by respondent type (self or caregiver), with weighting used to address differences in gender balance and SMA treatment status between the survey sample and U.S.-based SMA community estimates. Variables included in regression models included the three outcome variables along with gender, age at the time of the survey, race, income, SMA type, mobility, SMA drug treatment status, whether the affected individual had an in-person doctor's visit in the past year, and history of mental illness (anxiety or depression). Variables included in the unweighted regression models were tested for collinearity, and no significant collinearity was observed.

## Results

Sample characteristics are presented in *Table 1*. Compared with internal estimates for the U.S.-based SMA community, the original survey sample was more heavily female, more likely to have been treated with an SMA drug, and over-represented individuals who identify as white/Caucasian. The sample was also more highly educated, had higher household incomes, and was more likely to be insured than what would have been expected for the SMA community overall. Results that follow reflect the application of weights to account for differences in gender and treatment status.

**Table 1.** Characteristics of Spinal Muscular Atrophy-Affected Individuals Reflected in Sample

DEMOGRAPHIC CHARACTERISTICS		UNWEIGHTED		WEIGHTED		SMA-RELATED CHARACTERISTICS		UNWEIGHTED		WEIGHTED	
		***n***	%	***n***	%			***n***	%	***n***	%
Respondent	Caregiver	265	57.2	242	52.3	SMA type	Type 1	126	27.2	104	22.4
	Self	198	42.8	221	47.7		Type 2	192	41.5	177	38.2
Gender	Male	180	38.9	235	50.8		Type 3	128	27.7	147	31.7
	Female	281	60.7	226	48.8		Type 4	8	1.7	16	3.4
	Prefer not to answer	2	0.4	2	0.5		Unknown	9	1.9	20	4.4
Age	Average Years [Range]	21.2 [0, 76]		24.3 [0, 76]		Current maximum mobility	Non-sitter	85	18.4	84	18.1
Race	White	386	83.4	392	84.7		Sit	91	19.7	77	16.7
	Non-White	69	14.9	62	13.3		Stand	26	5.6	26	5.6
	Unknown	8	1.7	9	1.9		Walk	88	19.0	74	15.9
Education of affected individual	No Education	98	21.2	83	17.9		Unknown	173	37.4	202	43.7
	HS or Less	177	38.2	173	37.3	SMA drug treatment status	Untreated	47	10.2	146	31.5
	Some College or Associates	61	13.2	70	15.1		Treated	380	82.1	246	53.2
	Bachelors or Greater	127	27.4	138	29.8		Unknown	36	7.8	71	15.3
Household income	Under $20,000	76	16.4	79	17.0						
	$21,000–$40,000	56	12.1	57	12.3	General health and health care utilization		n	%	n	%
	$41,000–$70,000	77	16.6	73	15.8	Health rating^*^	Excellent	97	21.0	81	17.6
	$71,000–$100,000	92	19.9	91	19.6		Very good	190	41.0	170	36.6
	$101,000–$150,000	51	11.0	47	10.1		Good	110	23.8	108	23.4
	$151,000–$200,000	20	4.3	15	3.4		Fair	38	8.2	52	11.2
	Greater than $200,000	29	6.3	22	4.8		Poor	3	0.7	5	1.2
	Don't know	13	2.8	19	4.0		Unknown (Missing)	25	5.4	47	10.1
	Prefer not to answer	49	10.6	61	13.1	History of mental illness^**^	Yes	129	27.9	142	30.6
English primary language at home	No	15	3.2	11	2.4		No	319	68.9	304	65.6
	Yes	446	96.3	451	97.3		Unknown (Most Missing)	15	3.2	17	3.8
	Unknown (Missing)	2	0.4	1	0.3	In-person doctor's visit in past year	Yes	377	81.4	362	78.1
Insurance category	Any Public Insurance	310	67.0	318	68.6		No	84	18.1	99	21.3
	Private Insurance Only	92	19.9	77	16.6		Unknown (Missing)	2	0.4	3	0.6
	Military Service-Related	6	1.3	6	1.3						
	No Insurance	4	0.9	5	1.0						
	Unknown	15	3.2	21	4.5						
	Other	36	7.8	37	8.0						

^*^ Drawn from Health Utilities Index (HUI) data. The HUI is only validated for ages five and above.

^**^ Depression or anxiety.

SMA, spinal muscular atrophy.

As reflected in *Table 2*, about four-fifths of respondents had used telemedicine at least once. Before weighting, individuals represented by self-completed surveys were more likely to have any experience with telemedicine than individuals represented by caregiver-completed surveys. After weighting on gender and drug treatment, self-completed survey responses reflected roughly equivalent likelihood of any experience with telemedicine as caregiver-completed responses; however, affected individuals reported having used telemedicine more frequently. Affected individuals generally reported higher levels of comfort with telemedicine as well as perceived effectiveness, before and after weighting. Chi square tests did not reveal any significant association between insurance type and the outcomes of prior use, comfort with, and perceived effectiveness.

**Table 2.** Prior Use of and Attitudes About Telemedicine

VARIABLE	UNWEIGHTED ANALYSIS							WEIGHTED ANALYSIS					
	ALL SURVEYS		SELF-COMPLETED		CAREGIVER COMPLETED		***p***-VALUE^*^	ALL SURVEYS		SELF-COMPLETED		CAREGIVER COMPLETED	
Prior use of telemedicine (*n* = 463)	*n*	%	*n*	%	*n*	%		*n*	%	*n*	%	*n*	%
Never	81	17.5	30	15.2	51	19.3	<0.05	99	21.5	50	22.8	49	20.2
Once or twice	177	38.2	63	31.8	114	43.0		154	33.3	56	25.4	98	40.4
Several times	205	44.3	105	53.0	100	37.7		210	45.3	114	51.8	95	39.3
Comfort with telemedicine (*n* = 463)	*n*	%	*n*	%	*n*	%		*n*	%	*n*	%	*n*	%
Very uncomfortable	38	8.2	10	5.1	28	10.6	<0.001	43	9.3	13	6.1	30	12.3
Uncomfortable	36	7.8	8	4.0	28	10.6		37	8.0	15	6.7	22	9.1
Neutral	114	24.6	37	18.7	77	29.1		124	26.7	54	24.2	70	29.0
Comfortable	144	31.1	62	31.3	82	30.9		126	27.3	56	25.5	70	28.9
Very comfortable	131	28.3	81	40.9	50	18.9		133	28.7	83	37.4	50	20.7
Effectiveness of telemedicine (*n* = 462)	*n*	%	*n*	%	*n*	%		*n*	%	*n*	%	*n*	%
Not at all effective	18	3.9	10	5.1	8	3.0	<0.001	29	6.4	18	8.2	11	4.7
Minimally effective	86	18.6	20	10.1	66	25.0		84	18.2	24	10.8	60	24.9
Moderately effective	176	38.1	69	34.9	107	40.5		182	39.5	91	41.4	91	37.7
Effective	129	27.9	64	32.3	65	24.6		116	25.1	56	25.5	60	24.7
Very effective	53	11.5	35	17.7	18	6.8		50	10.9	31	14.0	19	8.0

^*^ Reflects statistical significance of differences between the respondent types (self vs. caregiver) in unweighted analysis.

### Predictors of prior use, comfort, and perceived effectiveness

Results of the ordered logit regression models are presented in *Table 3*. Statistically significant findings are noted below along with the directions of associations observed; odds ratios and *p*-values are in *Table 3*. All regression model results presented in the text of this article reflect weighting and adjustment for covariates, and—in cases of categorial variables—are described relative to the omitted reference categories noted in *Table 3*. For stratified analyses comparing results from self-completed surveys with caregiver-completed surveys, see *Supplementary Tables S1-1–3*. Unweighted regression analysis results are presented in *Supplementary Table S2*.

**Table 3.** Results of Regressions to Identify Predictors of Prior Use, Comfort, and Perceived Effectiveness

INDEPENDENT VARIABLES	OMITTED REFERENCE CATEGORY	COMPARISON CATEGORY	OUTCOMES: ODDS RATIOS AND ***p***-VALUES					
			PRIOR USE (***n*** = 461)		COMFORT LEVEL (***n*** = 462)		PERCEIVED EFFECTIVENESS (***n*** = 461)	
			ODDS RATIO	***p*** > |z|	ODDS RATIO	***p*** > |z|	ODDS RATIO	***p*** > |z|
Survey respondent	Self	Caregiver	1.09	0.875	3.25	0.015	1.42	0.458
Gender	Female	Male	1.70	0.012	0.86	0.427	0.79	0.229
		Prefer not to answer	1.98	0.599	0.28	0.294	0.35	0.360
Age at time of survey			1.01	0.168	0.99	0.423	0.99	0.522
Race	White	Non-White	1.40	0.257	0.59	0.046	0.67	0.139
		Unknown	0.65	0.509	0.66	0.460	0.82	0.756
Income			1.14	0.001	1.02	0.530	1.00	0.985
SMA type	Type 1	Type 2	0.59	0.060	1.35	0.228	2.11	0.003
		Type 3	0.34	0.002	1.38	0.284	2.03	0.019
		Type 4	0.67	0.526	1.43	0.510	1.26	0.671
		Unknown	3.01	0.121	1.67	0.275	2.11	0.160
Current maximum mobility	Nonsitter	Sit	1.11	0.749	1.10	0.760	0.55	0.048
		Stand	1.55	0.338	0.87	0.757	0.47	0.070
		Walk	1.40	0.344	0.60	0.103	0.25	<0.001
		Unknown	1.48	0.447	0.55	0.191	0.54	0.159
Treated with SMA drug	Untreated	Treated	1.97	0.014	1.32	0.237	2.26	0.001
		Unknown	1.85	0.083	0.89	0.726	1.48	0.224
In-person doctor's visit in Past Year	No	Yes	1.02	0.951	1.04	0.860	0.77	0.270
		Unknown (Missing)	0.38	0.386	0.012	0.006	38.35	0.025
History of mental illness	No	Yes	2.33	<0.001	1.21	0.373	1.35	0.171
		Unknown (Most Missing)	2.90	0.090	1.39	0.512	3.14	0.020
Prior use of telemedicine	No, never	Yes, but only once or twice			2.12	0.002	2.27	0.001
		Yes, several times			4.77	<0.001	4.83	<0.001
Comfort with telemedicine	Neutral	Very uncomfortable	2.50	0.025			Not included in regression^*^	
		Uncomfortable	0.81	0.603				
		Comfortable	2.48	0.001				
		Very comfortable	4.85	<0.001				
Perceived effectiveness of telemedicine for SMA	Not at all effective	Minimally effective	3.26	0.019	Not included in regression^*^			
		Moderately effective	3.41	0.012				
		Effective	3.83	0.010				
		Very effective	6.99	0.002				

Table includes results of ordered logit models with prior use, comfort level, and perceived effectiveness as outcome variables. All analyses reflect the application of weights to address differences in gender balance and SMA treatment status between the survey sample and U.S.-based SMA community estimates. Odds ratios with *p*-values <0.05 are highlighted along with corresponding *p*-values.

^*^ Perceived effectiveness is excluded from the model for comfort because initial modeling with perceived effectiveness included as an independent variable produced exceedingly high odds ratios, and comfort was excluded from the perceived effectiveness model for the same reason. These results led to the conclusion that the variables may be too closely related and should be excluded from each other's models.

Factors predicting greater likelihood of prior telemedicine use included male gender, increasing income, having received drug treatment for SMA, history of mental illness, and all non-neutral responses regarding comfort and perceived effectiveness, except “uncomfortable.” Having Type 3 SMA decreased the likelihood of telemedicine use compared to having Type 1.

Caregiver respondent type and prior use of telemedicine predicted increased comfort levels, with more frequent use having a more significant effect. Lack of information about whether a respondent had an in-person doctor's visit in the past year predicted lower comfort levels.

Finally, greater perceived effectiveness was predicted by an individual having Type 2 or 3 SMA, having received an SMA drug treatment, unknown mental health history, missing information about whether the survey subject had an in-person doctor's visit in the past year, and prior use of telemedicine. Being able to sit and walk predicted lower levels of perceived effectiveness.

When responses were stratified by respondent type, predictors varied across groups. While these results are not discussed in detail in this study, they are presented in *Supplementary Table S2* and are important because more factors emerged as statistically significant predictors of use, comfort, and perceived effectiveness for individuals who completed the survey themselves than for caregiver-completed surveys. Of particular note, higher levels of respondent education—which was included in the stratified analysis for self-completed surveys, but was not included in the full-group analysis because caregiver education was not captured in the survey—predicted increased likelihood of prior use, increased comfort, and increased perceived effectiveness for affected individuals.

## Discussion and Conclusions

These findings provide new perspective on telemedicine use within the SMA community. They reveal widespread uptake of telemedicine and illustrate varied perspectives on comfort and effectiveness, which are interrelated with demographic factors, health status, and health care utilization. While some of these associations seem logical, others will require additional examination. For instance, the finding that males are more likely than females to have had experience with telemedicine in the all-respondents model as well as the stratified models for affected individuals and caregivers raises important questions about why those differences exist, and whether they are a matter of personal preference, access to technology, or other factors. In addition, the relationships between type, mobility, and perceived effectiveness are surprising: it seems counterintuitive that having Type 2 and 3 SMA, which indicate greater maximum motor function, would predict greater perceived effectiveness when increased mobility predicts reduced perceived effectiveness.

These findings raise important considerations for use of telemedicine. In particular, the variability in experience and perspectives on telemedicine—as well as the observed associations between these outcomes and other factors such as the relationship to the affected individual (whether a caregiver or the individual themself), demographics, and health-related variables—suggests that there may be value in initiating level-setting conversations about expectations, advantages, and drawbacks surrounding telemedicine. Clinicians may consider proactively asking about how comfortable patients and caregivers feel with telemedicine before it is used or at the first virtual encounter, to address patient concerns and ensure that patients feel they are receiving an appropriate level of care. This may be especially relevant in circumstances where remote care options are needed and/or individuals are inexperienced or uncomfortable with telemedicine.

It is worth considering the numerous potential barriers to effective telemedicine use that may affect its utility, such as lack of access to technology and high-speed internet, as well as the relationships between demographics and use of telemedicine. For instance, female gender and lower income have both been associated with decreased use of telemedicine in other research; these are relationships that deserve further exploration and may hint at access barriers.^25^

This research has several limitations. First, the survey did not provide a specific definition for telemedicine, making it possible that respondents interpreted the term differently. From discussions with clinicians, clinical research coordinators, and caregivers, it is believed that most community members are likely to share a common understanding of telemedicine as a virtual meeting that occurs over a web-based video platform.

Second, selection bias is a concern. Analysis of survey characteristics revealed several differences between the survey sample and the U.S.-based SMA community. In addition, rurality was not included in the regression analyses due to survey data constraints, and may be an important missing factor. While these concerns were partially addressed by weighting, they limit the generalizability of these findings.

Because data on drug treatments were limited to whether an individual had received a drug treatment or not, associations between specific drugs and telemedicine outcomes could not be analyzed. Among other reasons, this is important because treatments are causing shifts in how SMA is classified and the outcomes and expectations of individuals with SMA.

More globally, the regression results are subject to the proportional odds assumption. There are also limitations associated with the self-reported nature of the data. These include recall bias and social desirability bias, which may be especially likely to affect questions such as those on mental health status. In addition, there are a number of important differences between caregiver-reported data and self-reported data, which raise questions about whether, at least for teenagers and adults, different answers might have been received from the affected individuals themselves. The fact that the survey was administered online may have also biased the respondent pool toward individuals who are comfortable with technology, which may affect attitudes about telemedicine.

This research represents an important first step in better understanding patient and caregiver use of and attitudes toward telemedicine use within the SMA population. It builds on prior qualitative research by providing perspective derived from a much larger set of patients and caregivers than prior studies that relied on focus groups.^9^ This insight is important because of recent expansion of telemedicine use and potential utility of telemedicine in care and research for SMA. Future research may build upon this work by seeking information from a larger, more representative sample. In addition, it would be worthwhile to seek more information about why individuals feel certain ways about telemedicine. These combined insights may facilitate more effective utilization of telemedicine, for delivery of care that is sensitive to the disease being treated as well as to patients and caregivers themselves.

## Supplementary Material

Supplemental data

Supplemental data
