# Supplementary material for: Telemedicine Use, Comfort, and Perceived Effectiveness in the Spinal Muscular Atrophy Community
Source: Telemed J E Health. 2024 Feb 7;30(2):536–44. doi: 10.1089/tmj.2023.0293 (PMC10877388; doi:10.1089/tmj.2023.0293)
Supplement: Supplemental data [file Suppl_TableS1.docx]

| Table S1-1. Predictors of Prior Telemedicine Use, Stratified by Respondent* | | | | | | | |
| --- | --- | --- | --- | --- | --- | --- | --- |
| Independent Variables | Omitted Reference Category | Comparison Category | Self-Completed Surveys (n=197) | | | Caregiver-Completed Surveys (n=264) | |
|  |  |  | Odds Ratio | P>\|z\| | Odds Ratio | | P>\|z\| |
| Gender | Female | Male | 3.26 | 0.010 | 1.95 | | 0.014 |
|  |  | Prefer not to answer | 6.53 | 0.274 |  | |  |
| Age at Survey |  |  | 1.02 | 0.154 | 1.00 | | 0.884 |
| Race | White | Non-White | 0.68 | 0.516 | 1.30 | | 0.487 |
|  |  | Unknown | 0.79 | 0.872 | 0.63 | | 0.574 |
| Education | High school or less | Some College or Associates | 3.66 | 0.033 |  | |  |
|  |  | Bachelors or Greater | 2.63 | 0.063 |  | |  |
| Income |  | Income | 1.23 | 0.003 | 1.06 | | 0.349 |
| SMA Type | Type 1 | Type 2 | 0.46 | 0.443 | 0.77 | | 0.440 |
|  |  | Type 3 | 0.25 | 0.168 | 0.24 | | 0.002 |
|  |  | Type 4 | 0.73 | 0.795 |  | |  |
|  |  | Unknown | <0.01 | 0.011 | 59.81 | | 0.002 |
| Current Maximum Mobility | Non-Sitter | Sit | 0.44 | 0.530 | 1.10 | | 0.784 |
|  |  | Stand |  |  | 1.70 | | 0.268 |
|  |  | Walk | 0.35 | 0.591 | 1.14 | | 0.738 |
|  |  | Unknown | 1.56 | 0.710 | <0.01 | | 0.981 |
| SMA Drug Treatment | Untreated | Treated | 2.11 | 0.079 | 1.46 | | 0.516 |
|  |  | Unknown | 5.55 | 0.032 | 1.64 | | 0.430 |
| In Person Doctor Visit in Past Year | No | Yes | 0.80 | 0.575 | 0.57 | | 0.241 |
|  |  | Unknown (Missing) | 0.32 | 0.450 |  | |  |
| History of Mental Illness | No | Yes | 3.89 | 0.002 | 1.87 | | 0.089 |
|  |  | Unknown (Most Missing) | 4.76 | 0.193 | 2.52 | | 0.307 |
| Comfort with Telemedicine | Neutral | Very uncomfortable | 4.40 | 0.108 | 1.11 | | 0.842 |
|  |  | Uncomfortable | 0.19 | 0.110 | 0.99 | | 0.986 |
|  |  | Comfortable | 3.47 | 0.013 | 1.38 | | 0.408 |
|  |  | Very comfortable | 12.60 | <0.001 | 1.21 | | 0.698 |
| Perceived effectiveness of telemedicine | Not at all effective | Minimally effective | 6.69 | 0.046 | 4.58 | | 0.101 |
|  |  | Moderately effective | 2.46 | 0.293 | 13.06 | | 0.005 |
|  |  | Effective | 6.25 | 0.042 | 9.99 | | 0.015 |
|  |  | Very effective | 3.73 | 0.212 | 27.29 | | 0.002 |
| *Analyses reflect the application of weights to address differences in gender balance and SMA treatment status between the survey sample and US-based SMA community estimates. Odds ratios with p-values less than 0.05 are highlighted along with corresponding p-values. | | | | | | | |

| Table S1-2. Predictors of Comfort with Telemedicine, Stratified by Respondent* | | | | | | | | | | | |
| --- | --- | --- | --- | --- | --- | --- | --- | --- | --- | --- | --- |
| Independent Variables | Omitted Reference Category | Comparison Category | | Self-Completed Surveys (n=197) | | | | Caregiver-Completed Surveys (n=265) | | | |
|  |  |  |  | Odds Ratio | | P>\|z\| | | Odds Ratio | | P>\|z\| | |
| Gender | Female | Male | | 0.57 | | 0.112 | | 0.92 | | 0.706 | |
|  |  | Prefer not to answer | | 0.21 | | 0.319 | |  | |  | |
| Age at Survey |  |  | | 0.98 | | 0.028 | | 1.03 | | 0.141 | |
| Race | White | Non-White | | 0.60 | | 0.292 | | 0.53 | | 0.045 | |
|  |  | Unknown | | 0.26 | | 0.226 | | 0.94 | | 0.936 | |
| Education | High school or less | Some College or Associates | | 2.82 | | 0.027 | |  | |  | |
|  |  | Bachelors or Greater | | 2.83 | | 0.017 | |  | |  | |
| Income |  | Income | | 1.01 | | 0.857 | | 1.03 | | 0.558 | |
| SMA Type | Type I | Type II | | 1.44 | | 0.578 | | 0.96 | | 0.894 | |
|  |  | Type III | | 1.53 | | 0.513 | | 0.87 | | 0.739 | |
|  |  | Type IV | | 1.97 | | 0.402 | |  | |  | |
|  |  | Unknown | | 4.64 | | 0.164 | | 1.28 | | 0.665 | |
| Current Maximum Mobility | Non-Sitter | Sit | | 1.79 | | 0.644 | | 1.25 | | 0.466 | |
|  |  | Stand | |  | |  | | 1.48 | | 0.375 | |
|  |  | Walk | | 0.35 | | 0.568 | | 0.75 | | 0.382 | |
|  |  | Unknown | | 0.42 | | 0.434 | | 1.86 | | 0.558 | |
| SMA Drug Treatment | Untreated | Treated | | 1.65 | | 0.136 | | 0.65 | | 0.351 | |
|  |  | Unknown | | 1.99 | | 0.246 | | 0.32 | | 0.028 | |
| In Person Doctor Visit in Past Year | No | Yes | | 0.93 | | 0.810 | | 1.10 | | 0.837 | |
|  |  | Unknown (Missing) | | 0.004 | | 0.002 | |  | |  | |
| History of Mental Illness | No | Yes | | 0.77 | | 0.463 | | 0.99 | | 0.973 | |
|  |  | Unknown (Most Missing) | | 1.62 | | 0.497 | | 1.05 | | 0.955 | |
| Prior Use of Telemedicine | No, never | Yes, but only once or twice | | 4.06 | | 0.002 | | 1.32 | | 0.384 | |
|  |  | | Yes, several times | | 13.16 | | <0.001 | | 1.97 | | 0.048 |
| *Analyses reflect the application of weights to address differences in gender balance and SMA treatment status between the survey sample and US-based SMA community estimates. Odds ratios with p-values less than 0.05 are highlighted along with corresponding p-values. | | | | | | | | | | | |

| Table S1-3. Predictors of Perceived Effectiveness, Stratified by Respondent* | | | | | | | | | | |
| --- | --- | --- | --- | --- | --- | --- | --- | --- | --- | --- |
| Independent Variables | Omitted Reference Category | Comparison Category | | Self-Completed Surveys (n=197) | | | Caregiver-Completed Surveys (n=264) | | | |
|  |  |  |  | Odds Ratio | P>\|z\| | | Odds Ratio | | P>\|z\| | |
| Gender | Female | Male | | 0.34 | 0.003 | | 0.93 | | 0.749 | |
|  |  | Prefer not to answer | | 0.28 | 0.300 | |  | |  | |
| Age at Survey |  |  | | 0.99 | 0.299 | | 1.02 | | 0.335 | |
| Race | White | Non-White | | 0.76 | 0.585 | | 0.71 | | 0.314 | |
|  |  | Unknown | | 0.46 | 0.528 | | 0.97 | | 0.968 | |
| Education | High school or less | Some College or Associates | | 3.75 | 0.007 | |  | |  | |
|  |  | Bachelors or Greater | | 4.54 | 0.001 | |  | |  | |
| Income |  |  | | 1.06 | 0.299 | | 0.95 | | 0.340 | |
| SMA Type | Type 1 | Type 2 | | 12.81 | <0.001 | | 1.43 | | 0.229 | |
|  |  | Type 3 | | 7.18 | 0.003 | | 2.30 | | 0.043 | |
|  |  | Type 4 | | 4.89 | 0.052 | |  | |  | |
|  |  | Unknown | | 74.59 | <0.001 | | 0.83 | | 0.802 | |
| Current Maximum Mobility | Non-Sitter | Sit | | 0.46 | 0.519 | | 0.54 | | 0.050 | |
|  |  | Stand | |  |  | | 0.51 | | 0.111 | |
|  |  | Walk | | 0.04 | 0.037 | | 0.28 | | <0.001 | |
|  |  | Unknown | | 0.23 | 0.170 | | 1.19 | | 0.876 | |
| SMA Drug Treatment | Untreated | Treated | | 2.45 | 0.009 | | 2.27 | | 0.100 | |
|  |  | Unknown | | 1.40 | 0.567 | | 1.65 | | 0.372 | |
| In Person Doctor Visit in Past Year | No | Yes | | 0.73 | 0.320 | | 0.69 | | 0.402 | |
|  |  | Unknown (Missing) | | 45.73 | 0.032 | |  | |  | |
| History of Mental Illness | No | Yes | | 1.23 | 0.553 | | 0.91 | | 0.774 | |
|  |  | Unknown (Most Missing) | | 14.76 | <0.001 | | 1.07 | | 0.930 | |
| Prior Use of Telemedicine | No, never | Yes, but only once or twice | | 3.27 | 0.014 | | 1.64 | | 0.152 | |
|  |  | | Yes, several times | 8.95 | | <0.001 | | 3.46 | | 0.001 |
| *Analyses reflect the application of weights to address differences in gender balance and SMA treatment status between the survey sample and US-based SMA community estimates. Odds ratios with p-values less than 0.05 are highlighted along with corresponding p-values. | | | | | | | | | | |
