# Supplementary material for: Telemedicine Use, Comfort, and Perceived Effectiveness in the Spinal Muscular Atrophy Community
Source: Telemed J E Health. 2024 Feb 7;30(2):536–44. doi: 10.1089/tmj.2023.0293 (PMC10877388; doi:10.1089/tmj.2023.0293)
Supplement: Supplemental data [file Suppl_TableS2.docx]

| Table S2. Predictors of Prior Use, Comfort, and Perceived Effectiveness of Telemedicine (Unweighted Analysis) | | | | | | | | |  |
| --- | --- | --- | --- | --- | --- | --- | --- | --- | --- |
| Independent Variables | Omitted Reference Category | Comparison Category | Outcome Variables | | | | | | |
|  |  |  | Prior Use  (n=461) | | Comfort (n=462) | | Perceived Effectiveness  (n=461) | | |
|  |  |  | OR | P>\|z\| | OR | P>\|z\| | OR | P>\|z\| | |
| Respondent | Self | Caregiver | 1.32 | 0.590 | 2.38 | 0.070 | 1.37 | 0.490 | |
| Gender | Female | Male | 1.74 | 0.008 | 0.90 | 0.574 | 0.95 | 0.789 | |
|  |  | Prefer not to answer | 2.26 | 0.548 | 0.22 | 0.193 | 0.25 | 0.307 | |
| Age at Survey |  |  | 1.00 | 0.723 | 1.01 | 0.298 | 1.02 | 0.060 | |
| Race | White | Non-White | 1.26 | 0.389 | 0.99 | 0.966 | 0.94 | 0.819 | |
|  |  | Unknown | 0.89 | 0.860 | 0.51 | 0.284 | 0.44 | 0.264 | |
| Income |  |  | 1.03 | 0.468 | 1.07 | 0.072 | 1.00 | 0.917 | |
| SMA Type | Type 1 | Type 2 | 0.67 | 0.117 | 1.04 | 0.863 | 1.49 | 0.090 | |
|  |  | Type 3 | 0.36 | 0.002 | 1.02 | 0.939 | 0.90 | 0.708 | |
|  |  | Type 4 | 0.56 | 0.475 | 0.65 | 0.536 | 0.56 | 0.462 | |
|  |  | Unknown | 0.89 | 0.892 | 1.75 | 0.373 | 1.05 | 0.948 | |
| Current Maximum Mobility | Non-Sitter | Sit | 1.35 | 0.317 | 0.96 | 0.877 | 0.71 | 0.239 | |
|  |  | Stand | 1.48 | 0.388 | 0.89 | 0.792 | 0.68 | 0.335 | |
|  |  | Walk | 1.60 | 0.143 | 0.58 | 0.069 | 0.46 | 0.011 | |
|  |  | Unknown | 1.73 | 0.252 | 0.72 | 0.452 | 0.59 | 0.218 | |
| SMA Drug Treatment | Untreated | Treated | 1.75 | 0.118 | 1.56 | 0.161 | 2.13 | 0.022 | |
|  |  | Unknown | 1.77 | 0.236 | 1.01 | 0.991 | 1.44 | 0.427 | |
| In Person Doctor Visit in Past Year | No | Yes | 0.97 | 0.913 | 1.03 | 0.890 | 0.69 | 0.112 | |
|  |  | Unknown (Missing) | 0.14 | 0.162 | 0.09 | 0.074 | 7.05 | 0.206 | |
| History of Mental Illness | No | Yes | 1.80 | 0.012 | 1.19 | 0.396 | 1.37 | 0.136 | |
|  |  | Unknown (Most Missing) | 1.92 | 0.245 | 0.77 | 0.615 | 1.17 | 0.755 | |
| Prior Use of Telemedicine | No, never | Yes, but only once or twice |  |  | 2.04 | 0.004 | 2.33 | 0.001 | |
|  |  | Yes, several times |  |  | 4.09 | <0.001 | 5.10 | <0.001 | |
| Comfort with Telemedicine | Neutral | Very uncomfortable | 1.85 | 0.135 |  | | Not included in regression** | | |
|  |  | Uncomfortable | 0.94 | 0.871 |  |  |  |  |  |
|  |  | Comfortable | 2.30 | 0.002 |  |  |  |  |  |
|  |  | Very comfortable | 3.22 | <0.001 |  |  |  |  |  |
| Perceived Effectiveness of Telemedicine | Not at all effective | Minimally effective | 2.13 | 0.163 | Not included in regression** | |  | | |
|  |  | Moderately effective | 2.80 | 0.057 |  |  |  |  |  |
|  |  | Effective | 3.59 | 0.023 |  |  |  |  |  |
|  |  | Very effective | 7.39 | 0.002 |  |  |  |  |  |
| *Table includes results of ordered logit models with prior use, comfort level, and perceived effectiveness as outcome variables. Odds ratios with p-values less than 0.05 are highlighted along with corresponding p-values.  **Perceived effectiveness is excluded from the model for comfort because initial modeling with perceived effectiveness included as an independent variable produced exceedingly high odds ratios, and comfort was excluded from the perceived effectiveness model for the same reason. These results led to the conclusion that the variables may be too closely related and should be excluded from each other’s models. | | | | | | | | |  |
